# A novel tool for assessing and summarizing the built environment

**DOI:** 10.1186/1476-072X-11-46

**Published:** 2012-10-17

**Authors:** Gretchen L Kroeger, Lynne Messer, Sharon E Edwards, Marie Lynn Miranda

**Affiliations:** 1 Nicholas School of the Environment, Duke University, Box 90328, Durham, NC, 27708, USA; 2 School of Community Health, College of Urban and Public Affairs, Portland State University, PO Box 751, Portland, OR, 97207, USA; 3 Children’s Environmental Health Initiative, School of Natural Resources and Environment, University of Michigan, 2046 Dana Building, 440 Church St, Ann Arbor, MI 48109, USA; 4 Department of Pediatrics, University of Michigan, 2046 Dana Building, 440 Church St, Ann Arbor, MI 48109, USA

## Abstract

**Background:**

A growing corpus of research focuses on assessing the quality of the local built environment and also examining the relationship between the built environment and health outcomes and indicators in communities. However, there is a lack of research presenting a highly resolved, systematic, and comprehensive spatial approach to assessing the built environment over a large geographic extent. In this paper, we contribute to the built environment literature by describing a tool used to assess the residential built environment at the tax parcel-level, as well as a methodology for summarizing the data into meaningful indices for linkages with health data.

**Methods:**

A database containing residential built environment variables was constructed using the existing body of literature, as well as input from local community partners. During the summer of 2008, a team of trained assessors conducted an on-foot, curb-side assessment of approximately 17,000 tax parcels in Durham, North Carolina, evaluating the built environment on over 80 variables using handheld Global Positioning System (GPS) devices. The exercise was repeated again in the summer of 2011 over a larger geographic area that included roughly 30,700 tax parcels; summary data presented here are from the 2008 assessment.

**Results:**

Built environment data were combined with Durham crime data and tax assessor data in order to construct seven built environment indices. These indices were aggregated to US Census blocks, as well as to primary adjacency communities (PACs) and secondary adjacency communities (SACs) which better described the larger neighborhood context experienced by local residents. Results were disseminated to community members, public health professionals, and government officials.

**Conclusions:**

The assessment tool described is both easily-replicable and comprehensive in design. Furthermore, our construction of PACs and SACs introduces a novel concept to approximate varying scales of community and describe the built environment at those scales. Our collaboration with community partners at all stages of the tool development, data collection, and dissemination of results provides a model for engaging the community in an active research program.

## Background

A host of studies seek to analyze the relationship among various elements of the built environment (BE) and health outcomes [1-9] and outline strategies for addressing built environment-related disparities [10]. Associations have been demonstrated between measures of crime, neighborhood walkability, and neighborhood deprivation and health outcomes like obesity and adverse pregnancy events [11-20]. These studies employ a variety of methods to assess the BE, including resident surveys [21-24], objective social surveys [6,9,25,26], and systematic social observations (SSO) using objective raters to visually assess neighborhood conditions [7,8,24,27].

Here, we briefly describe general types of built environment assessment tools; a detailed review of previously used tools for assessing neighborhoods was conducted by Schaefer-McDaniel et al. [28]. Resident surveys, which directly question residents on their perception of neighborhood conditions, exposure to stress-inducing variables, or the presence of physical and social incivilities, are subjective and may introduce same-source bias, meaning both neighborhood conditions and health are reported by the same individual [1,5,29]. They do, however, provide a clear sense of how the residents themselves view the quality and potential health effects associated with certain elements of the local BE. Objective social surveys typically use administrative datasets, such as US Census data, to construct deprivation indices composed of social factors that are then linked with health outcomes [30-32]. The statistical approaches that underlie Census data are robust, but are limited by the frequency and geographic scale at which Census data are collected. Detailed Census data are only available every 10 years, with some data only accessible at large areal units such as Census block groups or tracts, and data from the annual American Community Survey are more limited in scope than the decennial Census. In addition, only limited social and housing data are available to explain conditions of the BE. Systematic social observations are detailed, objective assessments conducted by raters using, among other things, paper or video surveys in an area for a specified list of conditions – conditions which may be delineated by local community members or community groups, researchers, local agency officials, or, ideally, collaboratively among all interested parties. In most SSOs, a small sample of block faces (both sides of a street) is used to represent larger neighborhood environments [6].

Prior residential built environment research identifies certain domains, incivilities and territoriality, which are able to describe the contribution of specific features of neighborhood environments to community health [5,6,8,9,26]. Incivilities measure physical disorder (e.g., litter or graffiti) and social disorder (e.g., prostitution or drug use), while territoriality or defensible space consists of “markers which convey a nonverbal message of control, separation from outsiders, and investment in the locale” [5]. Indicators of physical disorder have typically been included in one domain, regardless of whether the disorder characterizes property grounds versus buildings or privately held versus publicly held property.

This project, the Community Assessment Project (CAP), was undertaken by the Children’s Environmental Health Initiative (CEHI) and arose from collaborations with community stakeholders in Durham, NC. The goals of the CAP were to: 1) develop a systematic and comprehensive residential BE assessment tool; 2) design and implement a field data collection protocol that vested the community in the success of the CAP; 3) build an integrated Geographic Information System (GIS) of CAP and Durham County data; 4) summarize BE data into meaningful indices that can be linked to health data; and 5) widely disseminate the results of the CAP for use by community stakeholders, such as neighborhood residents, non-profit organizations, police, or government officials.

This paper describes a novel methodology developed for use by researchers and community members to assess the residential BE systematically, quickly, and comprehensively. For our work, we define the residential built environment as the elements of the built environment to which a person is exposed when passing through a neighborhood or community, but excluding infrastructure. CEHI’s CAP is at the tax parcel-level - a tax parcel is a designated area of land whose boundaries are recognized for tax purposes (e.g., residential and commercial properties). CEHI’s CAP is also an on-foot assessment using a comprehensive list of variables describing the physical condition of both the buildings and the local landscape. The approach is easily implemented and replicated in urban environments, yet relatively low-cost, while leveraging geospatial information technology and engaging the community throughout the process.

## Methods

### Instrument development

#### ***Literature review***

As a first step in designing the methodology, a review of the literature on BE assessments, systematic social observation, and neighborhood measures and scales was conducted. Although we recognize that the built environment includes the physical conditions of the home and the condition and design of infrastructure, this assessment is limited to residential elements of the built environment. Findings and lessons from previous studies of the built environment guided the construction of our survey instrument [6,8,9,24-26]. The BE variables and domains described by these studies were evaluated for their current relevance and supplemented with input from community members (see Table 1).

**Table 1:** **Community Assessment Project** (**CAP**) **variables**

	**Built environment domain**					
**Source**	**Housing damage**	**Property disorder**	**Territoriality**	**Vacancy**	**Nuisances**	**Miscellaneous (no domain)**
Literature	· Boarded door	· Litter	· Security bars	· Occupied	· Drug paraphernalia	· Property type
				· Unoccupied	· Food garbage	
	· Holes in walls	· Garbage	· No trespassing sign		· Inoperable vehicle	· Property sub-type
	· Roof damagae	· Broken glass	· Security sign		· Dog waste	· Front entry type

	· Chimney damage	· Discarded furniture	· Fencing		· Discarded furniture	· Garden
	· Foundation damage	· Discarded appliances			· Discarded appliances	· Greenery
	· Entry damage	· Discarded tires			· Discarded tires	· “For sale” sign
	· Door damage	· Inoperable vehicle			· Condoms	· “For rent” sign
	· Peeling damage	· High weeds			· Cigarette butts	· Home repair
	· Fire damage	· Fencing damage			· Alcohol container	· New home construction
	· Boarded windows	· Graffiti (on private property)			· Clothes	· Peeling paint
	· Broken windows				· Broken glass	
					· High weeds	
					· Graffiti (on public spaces)	
Community	· Condemned	· Cars on lawn	· Barbed wire	· Demolished	· Shopping carts	· Eviction notice
		· No grass	· “Beware of dog” sign		· Tree debris	
		· Standing water			· Large trash	· Dog
					· Batteries	
					· Fallen wire	
					· Broken water meter cover	
					· Uncovered storm drain	
					· Baby diapers	
					· Construction debris	
					· Deep holes	
					· Standing water	
Project leaders	Other condition	Other nuisance (on private property)			Other nuisance (on public spaces)	· Padlocked
						· Driveway present
						· Fence material
						· Fenced area
						· Window A/C unit

This table lists each of the variables used in the assessment of parcels (n=53) and public spaces (n=26), as well as the built environment domain they describe and the source that motivated the inclusion of each variable.

#### ***Variable selection***

CEHI investigators solicited input from community members through a series of individual and group meetings with community leaders in order to identify BE conditions that were of greatest concern to residents. We developed a variable list based on the literature and then supplemented the variable list with identified and observable variables that represented community concerns. Table 1 lists the variables included in the CAP tool and indicates which variables were based on the literature, on discussions with the Durham community, or developed by project leaders based on observations in the field. Several variables are based on, but are more specific than, the literature. We focused our efforts on two types of properties: privately-owned properties and public spaces (e.g., parks and green spaces). For each property, we assessed land use type, occupancy status, and the physical conditions of the building exterior, lawn/outdoor property, nuisances, and evidence of territoriality. Nuisances, or physical incivilities, (e.g., cigarette butts and graffiti) are items in public spaces that could be considered public eyesores or obstructions and are typically associated with neighborhood disorder and increased crime rates or fear of crime [7,8,33-35]. Territoriality has been defined as “the presence of physical markers which carry non-verbal messages of ownership, monitoring and protection, and a separation between one’s self or family and ‘outsiders’” [7]. These physical markers may include fences erected around a property or “No Trespassing” signs posted on a property. The same set of variables was used for residential, commercial, and other property types. For public spaces, we assessed nuisances and the presence and condition of sidewalks. Furthermore, certain nuisances were assessed for both parcels and public spaces.

The preliminary variable list was piloted in neighborhoods within the project area which we anticipated would span the conditions likely to characterize Durham’s built environment. Conditions or items observed during the pilot study, but not included in the preliminary variable list, were documented and later added to the final variable list. In total, each parcel was assessed on 53 variables and public spaces were assessed on 26 variables. During the study, if a condition or nuisance was observed, but had no corresponding variable in the database, it was recorded in a text field for “other nuisances” or “other conditions”. Sidewalks were documented by drawing a line with multiple points, or vertices, located along that line which would allow for the curvature of the sidewalk. Each sidewalk segment was denoted as broken or unbroken and obstructed or unobstructed.

### Project area

**Figure 1:**
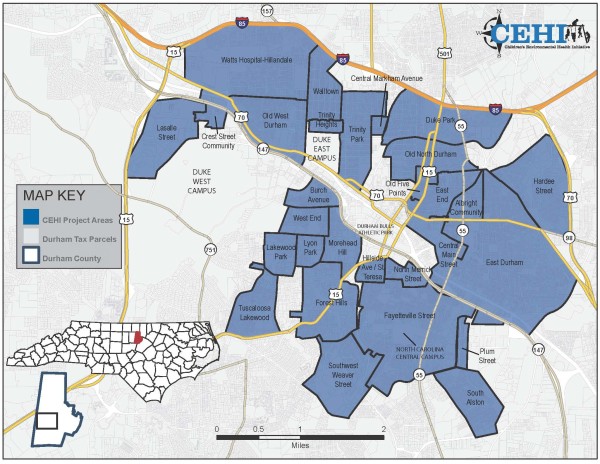
**CEHI Community Assessment Project (CAP) area.** This figure outlines each of the 29 neighborhoods in Durham, North Carolina composing the project area used for this study.

The CAP area is located in Durham, North Carolina, a city in which many non-governmental organizations, city and county departments, and academic institutions have conducted studies or programs related to neighborhood health, access to care, access to healthy food, and opportunities to engage in physical activity. However, no studies focusing on Durham have included an extensive assessment of the built environment – data that are valuable to the other efforts taking place in the city. The Durham is estimated to be home to 256,296 [36]. Within the county, 36.3 and 11.3 percent of the population are non-Hispanic black and Hispanic, respectively, and the median household income is $49,928 [36]. The study area focuses on Durham’s urban core and contains 29 defined neighborhoods (see Figure 1). Twenty-two of the neighborhoods are historic, with boundaries officially recognized by the City of Durham. Seven of the neighborhoods are established communities whose boundaries were approximated by CEHI personnel based on input from those communities.

### Supplemental administrative data

We obtained tax parcel data for 2007 from the Durham County Tax Assessor’s office and used parcel boundaries to build the database and to conduct the assessment. These data were also used to construct the tenure index, a measure of renter-occupied housing. To determine whether a property was owner or renter-occupied, we compared the geographic address of a parcel to the owner’s address. Using an algorithm that assessed the strength of the match between the parcel and owner address, we coded parcels as owner-occupied (addresses matched) or renter-occupied (addresses did not match). US Census 2000 block boundary files were acquired from the US Census Bureau so that data could be aggregated at the block level. Minor data management was required to correct misalignment of Census block boundaries and tax parcel boundaries. Crime data were obtained from the Durham Police Department Crime Analysis Unit and include reported crime incidents from 2006 – 2007 that are linked to the address at which the crime occurred. Each crime incident was geocoded to the street block or intersection at which the crime occurred. Crimes were then classified into major categories (violent, property, vice, theft, vehicular, and total) and aggregated to the Census block, resulting in counts of crime by type per block.

Tax parcel data were incorporated into the GIS database used for data collection and assigned fields for parcel ID and geographic address as unique identifiers. US Census blocks and crime data were incorporated into the GIS project after field work was complete. We aggregated the collected data and total counts of crime incidents to the block level.

### Data collection

#### ***Technology***

The software packages required to build the database include ESRI ArcGIS, Trimble GPS Analyst, ESRI ArcPad 7.0, and Trimble GPS Correct. ArcGIS is the desktop software used to build the database, GPS Analyst is an extension that enables databases for GPS, and ArcPad 7.0 was used for data collection and to record GPS coordinates for certain data types. The handheld GPS devices used to store the database and collect BE data were Trimble 2005 GeoXH units operating ArcPad 7.0 software. While we used the tool on high-end GPS units, efficient, lower-cost units are available and suitable for the assessment instrument that we built.

#### ***Database architecture***

The final variable list was organized into a GPS-enabled database ideal for editing in the field, which was created in ArcCatalog and readable in Microsoft Access. Separate spatial datasets, which could be overlaid within the GIS project, were created to hold data records for tax parcel centroids, nuisances, and sidewalks. Each spatial dataset included a table containing records for each spatial location (parcel centroid, nuisance, or sidewalk segment) in the project area and fields for relevant variables. Thus, each parcel centroid, nuisance point, and sidewalk could be edited independently. Records for nuisances and sidewalks were generated during the data collection process, while parcel records were preloaded into the GIS using a data layer provided by the Durham County Tax Assessor. In addition to the BE variables, each table includes longitude and latitude, date edited, data collector, and unique ID. Variables were assessed for their presence (1=Yes) or absence (0=No), as it was determined that using a scale would likely introduce inconsistency among our assessors. The database interface primarily consisted of drop-down menus with the default value set as “0 = No”, so that the underlying complexity of the data architecture was organized into a straightforward and user-friendly interface.

#### ***Training***

A CEHI staff member, the field team leader, managed a 5 person field team that included individuals of varying races/ethnicities and gender. Each field team member was trained for one week on the basics of GIS and the spatial analysis software package ArcGIS using instructional modules both from the training website for ESRI and those developed by CEHI’s spatial information technology training team. Field team members received instruction on using handheld GPS units. Following the GIS training, the interns participated in a second training period in which, over the course of a week, they received classroom and field instruction on the database used for the assessment. Topics included the structure of the database, the method of recording observations of variables, and the definitions of the variables included in the assessment tool. The field instruction took place in predetermined blocks in the study area to ensure variables would be coded properly and to strengthen inter-rater reliability.

#### ***Field protocol***

Prior to the execution of the community assessment, variables, methodology, and field protocol were tested during an eight month pilot study in 2007 using a team of 2–4 to assess parcels in all of the neighborhoods from the study area. After this pilot study, local neighborhood associations and other community groups, as well as the police department, were informed of when and where CEHI field team members would be working. Community partners were encouraged to relay word to community members about why the CAP was being undertaken and what to expect from the field team. All team members wore matching collared shirts with the CEHI logo, carried Duke University identification, and carried letters that provided a project description and contact information for both CEHI’s Director and Outreach Coordinator. These letters were distributed to any community member who approached the team during the assessment, and each field technician was coached in how to respond to public inquiry. As part of a safety protocol, all team members were always within sight of at least one other team member. Furthermore, all team members carried maps of the surrounding neighborhood blocks displaying locations of safe public buildings (e.g., stores, churches, and police stations) should the team need to exit an area rapidly (this proved useful when the field team inadvertently found itself in the middle of a SWAT team exercise!).

Of the 17,242 tax parcels within the 2008 study area, 598 were excluded due to unsafe roads (high traffic volume, speed limit > 30 mph, and no adequate shoulder or sidewalk for pedestrians) or lack of visibility from the public right of way. Thus, the on-foot, curbside assessment was completed for 16,644 tax parcels.

The team collected data from 7am – 1:30pm, Monday through Friday, May – August in 2008 and typically assessed about 1,500 properties per week. Several times a week, the field manager transferred spatial data from the database onto the handheld GPS units. This allowed the database to be taken out into the field, the tables opened, and the presence of specific BE variables documented. Upon completion of a predetermined area, approximately every 1 – 2 days, the field manager copied the populated data from the GPS units back into the database.

Parcels were assessed from all perspectives and angles possible by remaining on the sidewalk or on the street; at no time during assessment did data collectors trespass onto private property, nor were photographs of any sort taken at any time. Data management involved ensuring the data collector field was filled in for all data, entering the date of data collection, and checking the data for overlooked or twice-assessed parcels, nuisance points, and sidewalks.

One of the strengths of this project is that it was relatively low-cost to implement. The 5 person field team completed the training and field survey in a total of approximately 2,000 person–hours, and approximately 960 person hours were required from the project leader to complete data collection, management, and analysis. While CEHI already had the required computer assets, other sites interested in this approach may incur additional costs for the purchase of a computer, GIS software, mobile software, and GPS units.

#### ***Inter-rater reliability***

Inter-rater reliability (IRR), a measure of consistency or agreement between individual raters, was not calculated for data collection during 2008; however, since 2008 we have calculated IRR for a second round of CAP data collection during the summer of 2011. To calculate IRR in 2011, each field team member individually rated the same 50 parcels for the first several days of the assessment; thus, each property had 7 sets of ratings – 6 for the field team and team leader, the 7^th^ for the trainer. IRR was calculated with the “icc” (intraclass correlation) package in the R statistical program using the ratings for each property recorded by each assessor. This package computes intraclass correlation coefficients as an index of IRR . With 7 raters, the agreement across all variables was over 70% (95% confidence interval=0.684, 0.718), with an average agreement of 95% (95% confidence interval=0.945, 0.953), which is consistent with IRR and agreement in the literature [37]. The same supervisor conducted the training in 2008 and 2011, and the training materials and curriculum used were consistent across data collection periods; therefore, we are confident that the IRR for 2008 was of a similar strength.

#### ***Neighborhood definition***

**Figure 2:**
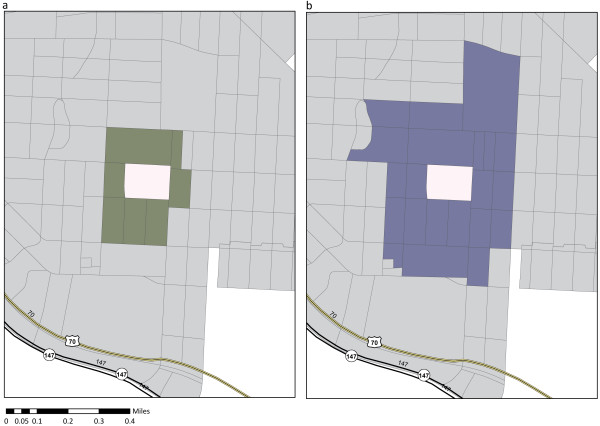
**Primary and secondary adjacency communities.** This figure illustrates the construction of Primary Adjacency Communities (PACs) in panel 2**a** and Secondary Adjacency Communities (SACs) in panel 2**b**.

There is a significant difference between the area represented by the smallest unit of aggregation, a block, and the next areal unit, a block group. Block groups do not necessarily represent community or neighborhood boundaries. Thus, we created primary adjacency communities (PACs) and secondary adjacency communities (SACs) to better understand neighborhood context and approximate the spatial scales that are likely to influence human health and quality of life. In order to determine PAC and SAC units of aggregation, we defined adjacent blocks as those blocks sharing a line segment (block boundary) and/or a vertex (block corner). A PAC was defined for each block, with each block’s PAC including itself and all adjacent blocks. Similarly, a SAC is cumulative and builds upon the PAC. A SAC was defined for each block, and comprises the PAC and all blocks adjacent to the PAC (see Figure 2). In contrast to pre-defined block groups, PACs and SACs act as moving windows – scoring each block with consideration of scores in adjacent blocks, even if these blocks fall in a different block group. PACs and SACs, therefore, may better describe the local area experience by residents of each Census block.

### Neighborhood indices characterizing the residential built environment

To create summary domains of the residential built environment, we examined the collected variables in order to identify which variables describe the same, or similar, features of the residential built environment. We then grouped variables likely to contribute to the same latent construct, meaning the variables are indicative of an unobservable factor likely to affect health rather than being expected to directly impact health. For example, a broken window and foundation damage both describe physical housing conditions, and while we would not expect a broken window or foundation damage individually to be associated with health, the underlying housing conditions these may highlight, especially when clustered, may be associated with health. Each variable was categorized into one of the following residential BE domains: housing damage (13 variables), property disorder (14 variables), measures of territoriality (6 variables), vacancy (3 variables), or nuisances (in public spaces only) (26 variables). Table 1 details which variables were assigned to each domain.

As this is the first tool to use such an exhaustive list of variables to characterize the residential built environment, original work on domain construction was required. As mentioned earlier, we expanded on the general domains of incivilities and territoriality from the existing literature to include the more specific domains of housing damage or disorder, property disorder, public nuisances, and territoriality. In addition, we developed 3 additional domains: tenure, vacancy, and crime. We note that: (1) each domain is unique and does not contain variables that might overlap with another domain; however, while certain BE features (i.e., “high weeds”) were assessed both in private and public spaces, the variables are distinct from each other; and (2) the specificity of the domains may help to explain which aspects of the residential built environment were most closely associated with health. The domains were constructed to enable investigators to describe the built environment in terms of “who” (vacant property containing no one, renter-occupied property, etc.) and “what” (damaged, disordered, and “claimed” territoriality) parcel conditions. While housing damage, property disorder, and nuisances may arguably belong in a larger physical incivilities domain, we felt it would be more informative to separate incivilities into three domains that would allow us to better identify which incivilities are associated with adverse health outcomes. It is difficult to determine if the effects observed between high rental neighborhoods and poor health outcomes is due to interpersonal factors (lack of stability in high rental neighborhoods) or to poor environmental quality (high rental neighborhoods tend to be more poorly maintained). Thus, one cannot determine which parts of the environment are contributing to the observed associations. However, with these data, if we observed association between vacancy and birth outcomes, but those properties were well maintained (not run down, as per the property disorder domain), we could hypothesize the association we observe has more to do with residential instability than presence of incivilities or poor quality spaces. By identifying which domains are driving the observed associations between the built environment and health, one would conclude that local government resources may be used more efficiently by targeting these residential BE features.

Parcel-level data (the directly observed CAP data and the tenure data collected from the tax-parcel database) were summed to the block-level to result in block-level counts of each variable for each domain. We constructed a vacancy index by identifying parcels that were unoccupied (unoccupied residential parcels, commercial parcels, religious institutions, or community properties, as well as vacant lots). A crime index was constructed using reported crime incidents for 2006 – 2007, differentiated by the charge (violent, theft, property, vice, vehicle, and total).

Construction of neighborhood-level indices began by aggregating the parcel-level indices to Census blocks to provide block-level totals for each index. There are 944 Census blocks located in the CAP area. The aggregation process was repeated at both the PAC and SAC level, resulting in each block containing a score for each index at the block, PAC, and SAC levels.

## Results

Of the 16,644 parcels assessed in 2008, 13,398 were residential, 681 were commercial, 1,253 were unoccupied or demolished empty lots (commercial or residential), 153 were faith or religious institutions, and 225 were community properties (such as community centers, cultural centers, and parks). The remaining 934 parcels fell under other categories. Of the 13,398 residential parcels, 505 contained apartments and 11,182 were single-family homes. The few remaining residential parcels were categorized as senior housing, care facilities, duplexes, multi-address homes, or other.

Table 2 details the prevalence of many variables for which each parcel was assessed. The parcel-level BE variables observed with the highest frequency included boarded windows (n=2,247), peeling paint (n=3,473), driveways (n=12,532), residential greenery (n=10,575), yard litter or garbage (n=5,116), high weeds or grass (n=2,090), security signage (n=4,051), and window AC units (n=2,271). Those that were not observed as often included roof damage (n=437), foundation damage (n=33), condemned residences (n=35), eviction notices (n=33), vegetable gardens (n=443), for sale signs (n=368), for rent signs (n=306), and graffiti (n=23).

**Table 2:** P**revalence of assessed characteristics**

**Parcel variables**	# **times observed**	**Public space nuisances**	# **times observed**
		Broken glass	4,171
Residential	13,398	Litter	11,970
· Single-family homes	11,182	High weeds/grass	2,025
· Apartments	505	Food garbage	5,511
· Senior housing, care facilities, duplexes, other	1,711	Cigarette butts/cartons	3,788
Commercial	681	Alcohol containers	1,260
Religious institution	153	Drug paraphernalia	13
Community	225	Graffiti	3
Unoccupied	1,253	Discarded appliances	61
Boarded windows	2,247	Discarded tires	66
Peeling paint	3,473	Condoms	82
Driveways	12,532		
Residential greenery	10,575		
Yard litter or garbage	5,116		
High weeds or grass	2,090		
Security signage	4,051		
Window AC units	2,271		
Roof damage	437		
foundation damage	33		
Condemned residence	35		
Eviction notice	33		
Vegetable garden	443		
For sale sign	368		
For rent sign	306		
Graffiti	23		

Table 2 summarizes the prevalence of the most commonly observed variables in the assessment.

There were a total of 31,652 nuisances observed in the public right-of-way. Those observed most frequently included broken glass (n=4,171), litter (n=11,970), high weeds or grass (n=2,025), food garbage (n=5,511), cigarette butts or cartons (n=3,788), and alcohol containers (n=1,260). Those observed with less frequency include drug paraphernalia (n=13), graffiti (n=3), discarded appliances (n=61), discarded tires (n=66), and condoms (n=82).

### Community descriptions

**Figure 3:**
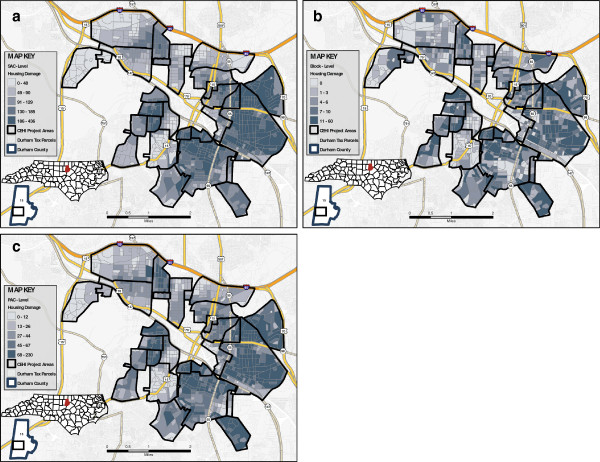
**Spatial patterns of neighborhood indices.** This figure demonstrates how the spatial pattern of one neighborhood index, housing damage, varies at each of the three units of aggregation: block (**a**), primary adjacency community (**b**), and secondary adjacency community (**c**).

While additional maps are available at the project website ( http://cehi.snre.umich.edu/projects/cap), here we provide an example showing how the housing damage index changes based on the levels of aggregation (see Figure 3a-c). These maps demonstrate the pattern in which the indices tend to be spatially distributed throughout neighborhoods. The block-level indices are characterized by a high degree of spatial variability, creating a mosaic pattern throughout the project area. The PAC- and SAC-level indices become less spatially variable as the indices are aggregated to a larger scale. At the block, PAC, and SAC levels, the strongest correlation was between property disorder and nuisances; however, nuisances, housing damage, and property disorder were all strongly correlated (see Table 3).

**Table 3:** Built environment indices correlations

		**Nuisances**	**Housing damage**	**Property disorder**	**Territoriality**	**Vacancy**	**Tenure**		**Crime**
Block-level									
	Nuisances	1.000							
	Housing Damage	0.804	1.000						
	Property Disorder	0.869	0.837	1.000					
	Territoriality	0.689	0.668	0.707	1.000				
	Vacancy	0.691	0.657	0.686	0.498	1.000			
	Tenure	−0.477	−0.378	−0.421	−0.066	−0.430	1.000		
	Crime	0.533	0.386	0.460	0.358	0.358	−0.294	0.190	1.000
PAC-level									
	Nuisances	1.000							
	Housing Damage	0.919	1.000						
	Property Disorder	0.944	0.915	1.000					
	Territoriality	0.751	0.757	0.773	1.000				
	Vacancy	0.803	0.765	0.772	0.572	1.000			
	Tenure	−0.648	−0.561	−0.571	−0.159	−0.631	1.000		
	Crime	0.656	0.498	0.609	0.447	0.469	−0.483	0.260	1.000
SAC-level									
	Nuisances	1.000							
	Housing Damage	0.952	1.000						
	Property Disorder	0.963	0.936	1.000					
	Territoriality	0.767	0.781	0.784	1.000				
	Vacancy	0.853	0.840	0.821	0.586	1.000			
	Tenure	−0.754	−0.694	−0.688	−0.281	−0.759	1.000		
	Crime	0.797	0.681	0.787	0.577	0.629	−0.649	0.333	1.000

Table 3 provides the correlation coefficients between indices at each of the three units of spatial aggregation: block, primary adjacency community (PAC), and secondary adjacency community (SAC).

The block-level indices for housing damage, property disorder, vacancy, nuisances, and crime were much higher in certain neighborhoods. Similarly, PAC- and SAC-level indices for these neighborhoods were much higher than other neighborhoods.

### Community outreach

As part of the CEHI outreach and education strategy, we designed and published a 20-page report that provides a brief description of the residential built environment, a discussion of its importance in community health, basic project information, and maps displaying the indices with explanations of why they may be of interest to and how they might be used by the Durham community. CEHI tailored the report style and design to maximize its usefulness to lay community members, researchers, and city leaders. Reports were distributed to county, state, and federal public health officials, as well as key stakeholders in Durham, NC, including religious leaders, community leaders, neighborhood organizations, and researchers. We also built a website ( http://cehi.snre.umich.edu/projects/cap) that provides project information and preformatted maps from the report that users can view and print individually.

## Discussion

Efforts to methodically assess the BE have generated a variety of valid methodologies, and this paper contributes to that literature. While previous studies relied on stratified sampling of Census geographies such as block groups and tracts [9,26,38], our tool allows for comprehensive assessment of properties within a large geographic area. Data at the block-level provides a general idea of BE conditions; however, these areal units may not reflect conditions of the larger community or neighborhood in which residents live and are engaged. Furthermore, we were able to build a database consisting of the residential built environment indicators from these field-tested and peer-reviewed studies that were relevant to Durham communities, while incorporating additional indicators that were of particular concern to residents or were observed during the pilot study. By replacing pen and paper instruments or video surveys (that can be upsetting to local community members) with a database edited in the field on GPS devices, raters were able to assess the built environment at a much quicker rate, thereby covering a greater geographic area very efficiently, and doing so in a way that furthered community interest in and acceptance of the work. For example, the field team put a human face on the research and answered many community questions as the data were being collected. This unique role supplemented the more formal community conversations that supported the study.

We also describe a novel method for combining residential built environment data into seven different domains that can easily be combined with secondary data that measure a community’s social environment: housing damage, property disorder, nuisances, territoriality, vacancy, tenure, and crime. Additionally, the units of aggregation described – block, PAC, and SAC – provide an alternative to traditional block-level analysis. This allows public health data to be linked to differing areal units, as appropriate for analysis. Miranda et al. demonstrate the ability to link these data to birth outcomes in Durham and tease out the association between the built environment and pregnancy outcomes [39].

While this study introduces a novel methodology to the BE assessment literature, it is not without limitations. Though objective, the tool described in this paper excludes any measure of residents’ perceptions of their neighborhood environment, which arguably moderates the impact of their neighborhood on their health. The instrument also does not measure social capital or community cohesion, which may mediate BE conditions. Furthermore, these data have the potential to vary seasonally. In North Carolina, data collected during summer months are likely to vary from those collected during fall or winter months due to seasonal patterns in resident behaviors and activities, as well changes in leaf litter and ground cover.

## Conclusions

This paper describes a tool used to assess the residential built environment at the tax parcel-level, as well as a methodology for summarizing the data into meaningful indices for linkages with health data. The key strength of this work is its easily-replicable design. With our assessment methodology, assessors collected exhaustive data characterizing the residential built environment within an urban context in a 13-week period, requiring approximately 2,000 person hours for a part-time field team, in addition to one full-time staff (including training time and the assessment). With a good training program and an experienced field team coordinator, this work can be accomplished by high school or college interns.

Furthermore, our construction of PACs and SACs to approximate varying scales of community and describe the BE at those scales introduces a novel concept; whereas studies similar in nature survey single, block-long street segments to proxy the BE at a larger spatial scale. Our collaboration with community partners at all stages of tool development, data collection, and dissemination of results, provides a model for engaging the community in spatially-based environmental health studies.

Furthermore, custom maps displaying these data have been developed to serve the needs of various community organizations, research groups, and local government agencies to inform health programs, community development initiatives, community-based participatory research, and community programs. Of significant achievement is a partnership formed between CEHI and the City of Durham’s Neighborhood Improvement Services (NIS) Department, wherein NIS will include block-level built environment data in a neighborhood index that will be used to identify and target high priority neighborhoods and communities for development and programs. These partnerships between CEHI and a variety of stakeholders demonstrate the utility of an exhaustive neighborhood assessment and the power of the data to inform programs, initiatives, and strategies at a local level.

## Abbreviations

GPS: Global Positioning System; PAC: Primary Adjacency Community; SAC: Secondary Adjacency Community; BE: Built Environment; SSO: Systematic Social Observations; CAP: Community Assessment Project; CEHI: Children’s Environmental Health Initiative; GIS: Geographic Information System; IRR: Inter-rater Reliability; NIS: Neighborhood Improvement Services.

## Competing interests

The authors declare they have no competing interests.

## Authors’ contributions

GK managed the field team, daily data collection, developed the methodology for summarizing the built environment data, and drafted the manuscript. LM provided expertise in built environment analyses and statistics, constructed and validated the domains, and helped draft the manuscript. SE reviewed the methodology and helped draft the manuscript. MLM conceived the study, participated in the design, oversaw the data collection, provided expertise in developing the methodology, and reviewed and edited the manuscript. All authors read and approved the final manuscript.
